# Physical frailty and pulmonary rehabilitation in COPD: a prospective cohort study

**DOI:** 10.1136/thoraxjnl-2016-208460

**Published:** 2016-06-13

**Authors:** Matthew Maddocks, Samantha S C Kon, Jane L Canavan, Sarah E Jones, Claire M Nolan, Alex Labey, Michael I Polkey, William D-C Man

**Affiliations:** 1 King's College London, Cicely Saunders Institute, London, UK; 2 NIHR Respiratory Biomedical Research Unit, Royal Brompton & Harefield NHS Foundation Trust and Imperial College, London, UK; 3 The Hillingdon Hospital NHS Foundation Trust, Middlesex, UK; 4 Harefield Pulmonary Rehabilitation Unit, Harefield Hospital, Middlesex, UK

**Keywords:** COPD epidemiology, Exercise, Pulmonary Rehabilitation

## Abstract

**Background:**

Frailty is an important clinical syndrome that is consistently associated with adverse outcomes in older people. The relevance of frailty to chronic respiratory disease and its management is unknown.

**Objectives:**

To determine the prevalence of frailty among patients with stable COPD and examine whether frailty affects completion and outcomes of pulmonary rehabilitation.

**Methods:**

816 outpatients with COPD (mean (SD) age 70 (10) years, FEV_1_% predicted 48.9 (21.0)) were recruited between November 2011 and January 2015. Frailty was assessed using the Fried criteria (weight loss, exhaustion, low physical activity, slowness and weakness) before and after pulmonary rehabilitation. Predictors of programme non-completion were identified using multivariate logistic regression, and outcomes were compared using analysis of covariance, adjusting for age and sex.

**Results:**

209/816 patients (25.6%, 95% CI 22.7 to 28.7) were frail. Prevalence of frailty increased with age, Global Initiative for Chronic Obstructive Lung Disease (GOLD) stage, Medical Research Council (MRC) score and age-adjusted comorbidity burden (all p≤0.01). Patients who were frail had double the odds of programme non-completion (adjusted OR 2.20, 95% CI 1.39 to 3.46, p=0.001), often due to exacerbation and/or hospital admission. However, rehabilitation outcomes favoured frail completers, with consistently better responses in MRC score, exercise performance, physical activity level and health status (all p<0.001). After rehabilitation, 71/115 (61.3%) previously frail patients no longer met case criteria for frailty.

**Conclusions:**

Frailty affects one in four patients with COPD referred for pulmonary rehabilitation and is an independent predictor of programme non-completion. However, patients who are frail respond favourably to rehabilitation and their frailty can be reversed in the short term.

**Key questions**

**What is the key question?**

- What is the prevalence of frailty in stable COPD, and does frailty affect the completion and outcomes of pulmonary rehabilitation?

**What is the bottom line?**

- Frailty affects one in every four patients with COPD entering pulmonary rehabilitation, is associated with favourable outcomes, but is also strong risk factor for non-completion.

**Why read on?**

- This is the first characterisation of the frailty phenotype in stable COPD and demonstrates that physical frailty is amenable to treatment with pulmonary rehabilitation.

## Introduction

Frailty describes a clinical syndrome characterised by multisystem decline that leads to reduced functional reserve and increased vulnerability to dependency or mortality following minor stressor events.^1^ It affects an estimated 1 in every 10 people aged over 65 years^2^ and is consistently associated with increased risk of falls, disability, hospitalisation and death.^3^ Although frailty is conventionally considered secondary to age-related decline, chronic disease(s) can accelerate the rate of decline and precipitate a frail state. In COPD, extrapulmonary manifestations include physical inactivity, muscle weakness, anorexia, osteoporosis and fatigue.^4^ Each of these systemic impacts of the disease is frequently observed in physical frailty.

The relevance of frailty to chronic respiratory disease has not been fully dissected. In retrospective cohort studies, self-reported frailty is more common in older people with COPD than without it, and markers of frailty have identified those at increased risk of subsequent hospital admission or death.^5^ ^6^ Few studies have examined the prevalence of frailty in respiratory disease using validated definitions. One notable exception is a report restricted to lung transplant candidates, among whom frailty was associated with increased risk of delisting or death.^7^ Identifying frailty earlier in the course of disease is important, as interventions may then be introduced to prevent functional decline, hospital admissions and/or death in those at high risk. Frailty may prove a valuable way of stratifying patients with COPD for future management as it accounts for multiple deficits that influence disease prognosis, for example, muscle weakness or physical inactivity,^8^ including deficits not considered by other syndromes or comorbidity indices.^9^ ^10^

Another important but unstudied topic is the interplay between frailty and pulmonary rehabilitation. Pulmonary rehabilitation is highly effective at improving symptom burden, physical function and health status, although patient response is heterogeneous.^11^ ^12^ Conceptually, pulmonary rehabilitation targets many components of frailty, including slowness,^13^ fatigue,^14^ weakness^15^ and physical inactivity,^11^ and provides a holistic approach to encourage self-management, disease education and behaviours to improve overall health. Pulmonary rehabilitation also targets dyspnoea, which may be a contributing factor to the development of frailty in people with chronic respiratory disease. Finally, some programmes incorporate falls prevention strategies, through balance training and education,^16^ again focusing on a frailty-related outcome. However, in the same way that frailty affects planned surgical management,^7^ the syndrome may prevent patients from engaging in pulmonary rehabilitation—a mainstay of disease management.^11^ The factors for heterogeneous adherence to pulmonary rehabilitation are widely debated,^17^ ^18^ but physical frailty is a plausible candidate given the close relationship with adverse outcomes in COPD^6^ ^7^ and other long-term conditions.^3^ If frailty does hinder completion of pulmonary rehabilitation, this would suggest a need to support patients who are frail with alternative or supplementary rehabilitation strategies.

Study objectives were to determine the prevalence of frailty among patients with stable COPD, describe the clinical characteristics of the COPD frailty phenotype and examine whether frailty affects completion and clinical outcomes of pulmonary rehabilitation. We hypothesised that frailty would independently predict non-completion of pulmonary rehabilitation.

## Methods

### Participants and design

Participants were recruited to this prospective cohort study from respiratory outpatient and pulmonary rehabilitation clinics at Harefield Hospital (Middlesex, UK) between November 2011 and January 2015. Eligible patients were aged 35 years or above, with a physician diagnosis of COPD consistent with the Global Initiative for Chronic Obstructive Lung Disease (GOLD) criteria^19^ and appropriate for a pulmonary rehabilitation referral. Exclusion criteria were an exacerbation in the previous 4 weeks that required a change in medication, a condition that might make moderate-intensity exercise unsafe, for example, unstable cardiac disease or a predominant neurological disability. Treating clinicians identified potentially eligible patients and offered a written information sheet. Referral criteria for pulmonary rehabilitation were able to walk at least 5 m, any degree of functional impairment secondary to dyspnoea (typically Medical Research Council (MRC) dyspnoea score 2 or more), no previous supervised pulmonary rehabilitation in previous 12 months and without unstable cardiovascular disease, in line with British Thoracic Society Quality Standards.^20^ Data for some participants (528/816, 64.7%) relating to sarcopenia, but not frailty, have been reported previously.^15^ All participants gave written informed consent in accordance with the principles of Good Clinical Practice and the Declaration of Helsinki, and the study was approved by the West London (11/H0707/2) and London Camberwell St Giles (11/LO/1780) Research Ethics Committees.

### Frailty assessment

Frailty was defined using the Fried phenotype model,^9^ which is well established and validated in large epidemiological studies.^3^ This model comprises five characteristics that reduce physiological reserve and precipitate a vulnerable state; shrinking (unintentional weight loss), exhaustion, low physical activity, slowness and weakness.^9^ Characteristics were assessed by weight loss history, two questions from the Center for Epidemiological Studies-Depression (CES-D) questionnaire (exhaustion),^21^ weekly self-reported energy expenditure using the modified Minnesota Leisure-Time Physical Activity Questionnaire (low physical activity),^22^ 4-m gait speed (4MGS, slowness)^23^ and handgrip dynamometry (weakness). Standardised criteria, derived from the original reference cohort,^9^ were used to define each characteristic as either present or absent for each patient (see online supplementary table S1), providing an ordinal score ranging 0–5. Patients with no criteria present were considered not-frail/robust, those meeting 1–2 criteria were considered prefrail and those with ≥3 criteria present were considered frail.

10.1136/thoraxjnl-2016-208460.supp1 Supplementary data

### Additional assessments

Bioelectrical impedance analysis (Quadscan 4000, Bodystat, Isle of Man, UK) was used to estimate skeletal muscle mass index (SMI).^24^ The presence of sarcopenia was defined according to the consensus European Working Group on Sarcopenia in Older People criteria.^25^ Quadriceps maximum voluntary contraction (QMVC) was measured using a fixed strain gauge,^26^ with weakness diagnosed according to healthy predicted values^27^ and sex-specific functionally relevant cut-points.^28^ Exercise performance was assessed using the incremental shuttle walk test (ISWT).^29^ Further measurements included evaluation of respiratory disability by MRC dyspnoea score, comorbidity burden using the age-adjusted Charlson Index,^30^ help with activities of daily care using the Katz Index,^31^ composite disease severity by the age, dyspnoea, and airflow obstruction (ADO) index,^32^ Hospital Anxiety and Depression Scale (HADS) and health status using the self-reported Chronic Respiratory Questionnaire (CRQ)^33^ and COPD Assessment Test (CAT).^34^

### Pulmonary rehabilitation

Pulmonary rehabilitation was an 8-week outpatient exercise and multidisciplinary education programme, comprising two supervised and at least one additional home-based session each week and organised according to the British Thoracic Society Quality Standards for Pulmonary Rehabilitation.^20^ Supervised sessions comprised 1 hour of exercise and 45 min of education. Exercise training was individualised and progressive. Initial walking speed prescription was at 80% of predicted peak oxygen consumption based on ISWT performance,^35^ while initial endurance cycling was set at a workload with the aim of patients completing 10 min of continuous training. Lower limb resistance training comprised 2 sets of 10 seated leg press repetitions, performed with an initial training load of 60% one-repetition maximum, as well as sit-to-stand, knee extension, hip flexion and hip abduction exercises with ankle weights. Upper limb resistance training comprised biceps curls, shoulder press and upright row with free weights. Education was delivered by a multidisciplinary team. Topics were chosen to develop patients' understanding and holistic management of their disease and included physical activity and exercise, medication use, diet, smoking cessation, coping strategies, as well as managing infections through early recognition, rescue medication and appropriate general practice/hospital presentation.

### Statistical analysis

Our sample size was based on the precision to which the overall prevalence of frailty could be estimated. Assuming it was within the range 10%–60%, prevalence could be estimated to within ±3.5% using 800 participants with a large sample normal approximation (nQuery Advisor V.6.0). Analyses were performed using SPSS (V.22, IBM, New York, USA) and graphs produced using Prism 5 (GraphPad Software, San Diego, California, USA). Data were presented as proportions (95% CIs) or mean (SD).

The prevalence of frailty was determined overall and then compared across groups according to age, GOLD spirometry stage, MRC dyspnoea score and age-adjusted Charlson comorbidity score using χ^2^ for trend. Baseline characteristics as well as pulmonary rehabilitation uptake, attendance and completion rates were compared across groups (not frail/robust, pre-frail, frail) using one-way analysis of variance or χ^2^ for trend with a Bonferroni correction applied to post hoc pairwise comparisons. Uptake was defined as the proportion of assessed patients who attended the first supervised session, adherence was assessed using the number of supervised sessions attended and completion was defined as the proportion of patients who had documented attendance at a minimum of eight supervised sessions, representing 50% attendance.^36^

Univariate logistic regression was used to assess the relationships between non-completion, frailty status (not or prefrail/frail) and candidate explanatory variables informed from existing literature and clinical judgement.^17^ ^18^ Variables associated with non-completion (p<0.15) were considered in a multivariate model. After checking for collinearity (r<0.75), we used a backwards conditional approach to retain variables in the model (p<0.10). Outcomes of rehabilitation were summarised as change pre-to-post rehabilitation for patients who completed as per the above definition. Outcomes were then compared across groups using analysis of covariance adjusting for age and sex. To control for Type I errors in view of multiple testing, we applied a Bonferroni correction to a significance level of 0.05 when comparing baseline patient characteristics and rehabilitation outcomes.

## Results

### Prevalence of frailty

**Figure 1:**
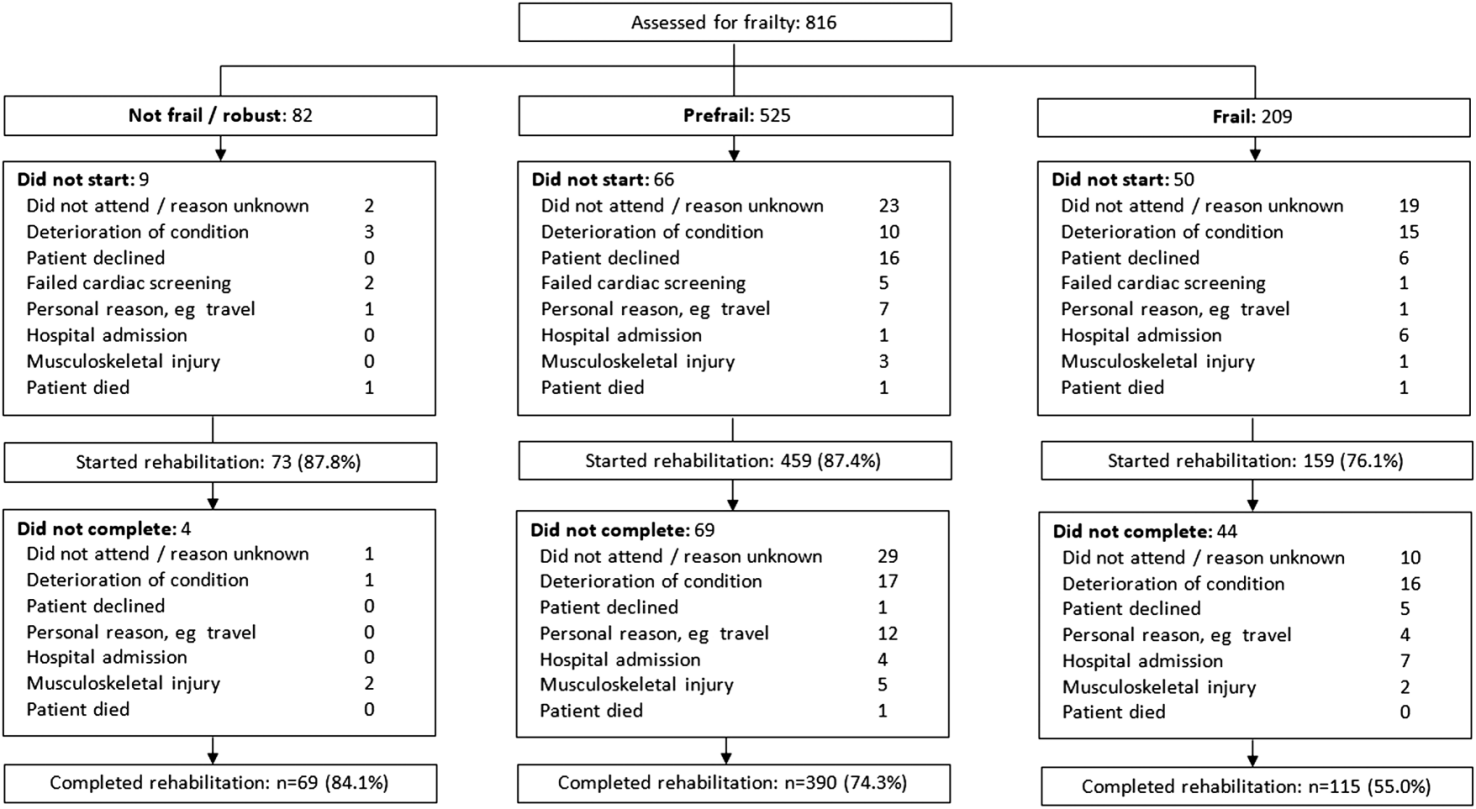
Profile showing recruitment, frailty status and flow of patients through the trial with reasons for non-uptake or non-completion of pulmonary rehabilitation.

**Figure 2:**
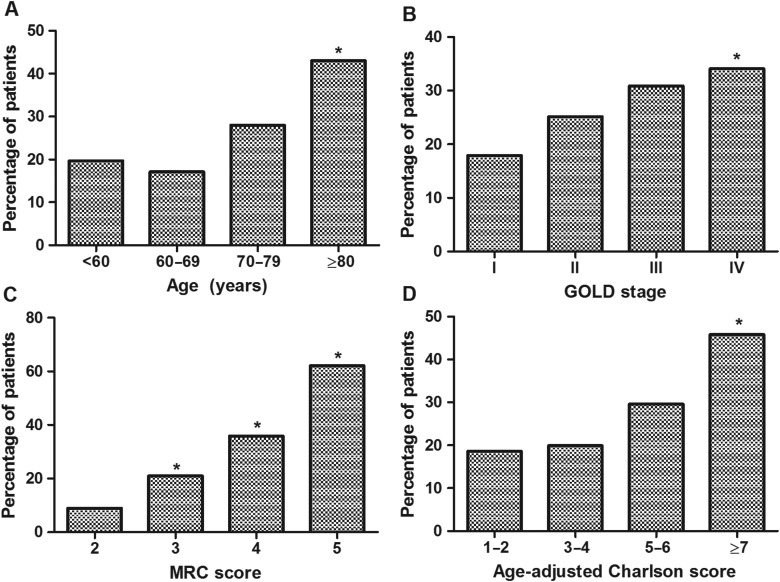
Prevalence of frailty in COPD according to age (A), GOLD spirometric stage (B), Medical Research Council (MRC) Dyspnoea score (C) and co-morbidity burden (D) (n=816). Between-group differences (p<0.01) compared with base group (far left) denoted by asterisk.

Eight-hundred and sixteen patients took part (484 men, mean (SD) age 70 (10) years, FEV_1_ 48.9 (21.0) % predicted, MRC score 3.3 (1.0)) (table 1). The overall prevalence of frailty was 25.6% (95% CI 22.7% to 28.7%), while 10.0% (95% CI 8.2% to 12.3%) of patients did not meet any of the frailty criteria and were considered robust (figure 1). Of the frailty characteristics, the exhaustion criterion was met by the largest proportion of patients (65.3%) followed by weakness, low physical activity and slowness. The unintentional weight loss criterion was met by fewest patients (14.2%) (table 1). Frailty tended to be more common among women than men (29.7% vs 22.8%, p=0.08). Prevalence increased statistically with increasing age (p<0.001), GOLD stage (p=0.01), MRC score (p<0.001) and comorbidity burden (p=0.004). There was a twofold increase in frailty prevalence among patients with GOLD stage IV as compared with stage I disease (34.7% vs 17.9%, p<0.001) and a threefold increase among patients with an MRC score of 5 as compared with a score of 3 (62.1% vs 21.0%, p<0.001; figure 2).

**Table 1:** Baseline characteristics and progression through pulmonary rehabilitation for the total cohort and stratified by frailty status

	All (n=816)	Not frail (n=82)	Prefrail (n=525)	Frail (n=209)	p Value
Age (years)	69.8 (9.7)	67.4 (8.1)	69.0 (9.6)	72.6 (10.0)*†	<0.001
Males, n (%)	484 (59.3)	49 (59.8)	324 (61.7)	110 (52.6)	0.08
Smoking status Current:former:never (%)	17.9:76.5:6.6	9.8:84.1:6.1	18.7:75.0:6.3	15.3:77.0:7.7	0.45
FEV_1_ % predicted	48.9 (21.0)	57.0 (22.4)	49.0 (20.8)	46.3 (20.1)*	<0.001
MRC score	3.3 (1.1)	2.4 (0.8)	3.2 (1.0)	4.0 (0.9)*†	<0.001
Age-adjusted Charlson Index	4.3 (1.6)	4.4 (1.6)	4.3 (1.6)	4.4 (1.6)	0.57
ADO score	4.9 (1.7)	3.6 (1.3)	4.7 (1.6)	6.0 (1.5)*†	<0.001
BMI (kg/m)	27.8 (6.7)	27.2 (5.2)	27.8 (6.5)	27.9 (7.6)	0.71
SMI (kg/m^2^)	8.47 (1.87)	8.6 (1.9)	8.6 (1.9)	8.1 (1.8)*†	0.002
Sarcopenia (%)	12.4 (10.3, 14.8)	1.2 (0.2, 6.6)	9.5 (7.3, 12.3)	23.9 (18.6, 30.1)	<0.001
Handgrip (kg)	27.0 (9.9)	33.0 (8.9)	28.3 (9.6)	21.3 (8.2)*†	<0.001
Peak QMVC (kg)	26.2 (10.0)	31.0 (10.1)	27.3 (9.6)	21.0 (9.0)*†	<0.001
QMVC % predicted	59.1 (20.0)	67.2 (20.6)	60.6 (20.0)	51.1 (16.9)*†	<0.001
Below QMVC cut-point (%)	25.9 (22.8, 29.2)	6.4 (2.8, 14.1)	21.4 (18.0, 25.3)	44.0 (36.8, 51.6)*†	<0.001
4MGS (m/s)	0.90 (0.24)	1.11 (0.21)	0.95 (0.19)	0.66 (0.20)*†	<0·001
ISWT (m)	222.3 (151.3)	375.4 (168.3)	245.1 (135.4)	105.0 (89.5)*†	<0.001
CRQ dyspnoea score	13.9 (5.6)	16.4 (5.9)	14.0 (5.7)	12.7 (5.0)*†	<0.001
CRQ fatigue score	13.4 (5.3)	17.6 (4.3)	13.8 (5.2)	10.9 (4.6)*†	<0.001
CRQ emotional score	30.6 (9.5)	36.1 (7.7)	31.3 (9.1)	26.6 (9.5)*†	<0.001
CRQ mastery score	17.6 (6.0)	21.7 (4.6)	18.0 (5.8)	15.0 (5.7)*†	<0.001
Self-reported weekly energy expenditure (kcal)	698.9 (1478.1)	1878.4 (1631.0)	1110.5 (1549.7)	257.2 (450.0)*†	<0.001
Self-reported time in moderate activity (min/week)	279.9 (431.1)	532.8 (491.7)	322.6 (465.0)	73.6 (1292)*†	<0.001
CAT score	20.7 (8.3)	13.3 (5.6)	20.2 (7.8)	25.0 (7.9)*†	<0.001
Katz score	5.7 (0.7)	5.9 (0.2)	5.8 (0.5)	5.4 (1.0)*†	<0.001
HADS anxiety	7.1 (4.6)	5.2 (3.4)	6.8 (4.4)	8.3 (5.2)*†	<0.001
HADS depression	6.6 (3.8)	4.1 (2.7)	6.3 (3.7)	8.2 (4.0)*†	<0.001
Self-reported hospital admission in previous year, n (%)	337 (41.3)	28 (34.1)	215 (41.0)	94 (45.0)*†	<0.001
Self-report number of exacerbations in previous year	2.8 (3.6)	2.1 (2.3)	2.8 (3.9)	3.1 (3.0)*	<0.001
Frailty characteristic (% meeting criteria)					
Unintentional weight loss	14.2 (12.0 to 16.8)	0	11.8 (9.3 to 14.9)	25.4 (19.9 to 31.7)	<0.001
Exhaustion	65.3 (62.0 to 68.5)	0	68.4 (64.3 to 72.2)	83.3 (77.6 to 87.7)	<0.001
Low physical activity	35.9 (32.7 to 39.3)	0	23.8 (20.4 to 27.6)	80.4 (74.5 to 85.2)	<0.001
Slow gait speed	24.4 (21.6 to 27.4)	0	9.1 (7.0 to 11.9)	72.2 (65.8 to 77.9)	<0.001
Weak handgrip strength	43.6 (40.3 to 47.1)	0	35.8 (31.8 to 40.0)	80.4 (74.5 to 85.2)	<0.001
Pulmonary rehabilitation					
Started (% of referred)	84.7 (82.0 to 87.0)	80.4 (94.1)	87.4 (84.3 to 90.0)	76.1 (69.9 to 81.4)	<0.001
Number of sessions attended	11.4 (4.2)	13.2 (3.0)	11.6 (4.1)	10.2 (4.7)	<0.001
Completed (% of starters)	83.1 (80.1 to 85.7)	94.5 (86.7 to 97.8)	82.8 (79.1 to 86.0)	72.3 (64.9 to 78.7)	<0.001
Completed (% of referred)	70.3 (67.1 to 73.4)	84.1 (74.7 to 90.5)	74.3 (70.4 to 77.8)	55.0 (48.2 to 61.6)	<0.001

Values are mean (SD) or proportions (95% CI) unless stated.

*Statistically different to non-frail group.

†Statistically different to prefrail group.

4MGS, 4-m gait speed; ADO, age, dyspnoea, and airflow obstruction; BMI, body mass index; CAT, COPD Assessment Test; CRQ, Chronic Respiratory Disease Questionnaire; HADS, Hospital Anxiety and Depression scale; ISWT, incremental shuttle walk test; kcal, kilocalorie; MRC, Medical Research Council; QMVC, quadriceps maximum voluntary contraction; SMI, skeletal muscle mass index.

### The COPD frailty phenotype

Patients who were frail with COPD had significantly reduced SMI but not body mass index as compared with prefrail or robust patients (table 1). Reduced physical function was evident beyond the characteristics used to define frailty, with reduced QMVC and ISWT performance, and a greater prevalence of sarcopenia (23.9% vs 9.5% in the prefrail group and 1.2% in the robust group, p<0.001). Patients who were frail reported a worse health status across all instruments and domains, and higher levels of anxiety and depression as compared with patients who were not-frail and prefrail (table 1). Among patients with QMVC measurements (n=707), almost three-quarters (73.1%) of patients who were frail had concurrent quadriceps weakness,^20^ while only 25.0% had concurrent sarcopenia. Similar proportions of patients who were frail had both (14.4%) or neither (16.3%) of these phenotypes.

### Engagement in pulmonary rehabilitation

Overall rates of programme uptake and completion for the cohort were 84.7% (95% CI 82.0% to 87.0%) and 70.3% (95% CI 67.1% to 73.4%), respectively, and mean (SD) attendance was 11.4 (4.2) of 16 supervised sessions (table 1). Rates were lowest in the frail group such that 55.0% of candidates completed rehabilitation, as compared with 74.5% of prefrail and 84.1% of not frail/robust candidates (p<0.001). In the univariate regression age, MRC score, FEV_1_% predicted, Charlson Index, ISWT, QMVC, ADO, CAT score, HADS anxiety and depression, and frailty status were associated with non-completion. Each of the frailty characteristics was individually associated with non-completion though the relationship was strongest when frailty status was considered overall (table 2). In the multivariate regression, frailty status, age, ISWT and CAT score were retained in the final model. Frailty was a strong independent predictor and being frail was associated with double the odds of non-completion; adjusted OR 2.20 (95% CI 1.39% to 3.46%), p=0.001 (table 2). When examining reasons for not taking up or completing a programme, proportionally more frail patients experienced deterioration in their condition or were admitted to hospital (figure 1). Among patients who were frail, baseline MRC score was higher in non-completers as compared with completers (4.3 vs 3.9, p=0.001) and there was weak evidence of additional functional impairment in non-completers (see online supplementary table S2).

**Table 2:** Univariate and multivariate logistic regression for variables associated with non-completion of pulmonary rehabilitation in patients with COPD (n=816)

Univariate	OR (95% CI)	p Value
Age	0.983 (0.967 to 0.998)	0.029
Sex	0.855 (0.628 to 1.164)	0.32
Current smoker (yes/no)	1.000 (1.000 to 1.000)	0.52
MRC	1.619 (1.388 to 1.887)	<0.001
FEV_1_ % predicted	0.995 (0.987 to 1.002)	0.15
GOLD stage	1.000 (1.000 to 1.000)	0.52
Age-adjusted Charlson Index	0.926 (0.849 to 1.019)	0.11
ISWT distance	0.996 (0.995 to 0.998)	<0.001
QMVC	0.986 (0.970 to 1.003)	0.12
ADO	1.122 (1.022 to 1.231)	0.015
CAT score	1.059 (1.038 to 1.080)	<0.001
HAD anxiety	1.061 (1.027 to 1.096)	<0.001
HAD depression	1.093 (1.051 to 1.137)	<0.001
Frailty characteristic		
Unintentional weight loss	1.578 (1.0044 to 2.385)	0.030
Exhaustion	1.615 (1.154 to 2.261)	<0.001
Low physical activity	2.334 (1.708 to 3.189)	<0.001
Slow gait speed	2.317 (1.655 to 3.244)	<0.001
Weak handgrip strength	1.258 (0.927 to 1.709)	0.141
Frailty status (not or pre-frail/frail)	2.699 (1.936 to 3.762)	<0.001

Multivariate	Adjusted OR (95% CI)	p Value
Age	0.964 (0.944 to 0.984)	0.001
ISWT distance	0.998 (0.997 to 1.000)	0.026
CAT score	1.024 (0.998 to 1.051)	0.07
Frailty status, yes/no	2.195 (1.392 to 3.463)	0.001

ADO, age, dyspnoea, and airflow obstruction; CAT, COPD Assessment Test; HADS, Hospital Anxiety and Depression scale; ISWT, incremental shuttle walk test; MRC, Medical Research Council; QMVC, quadriceps maximum voluntary contraction.

### Outcomes of pulmonary rehabilitation

**Figure 3:**
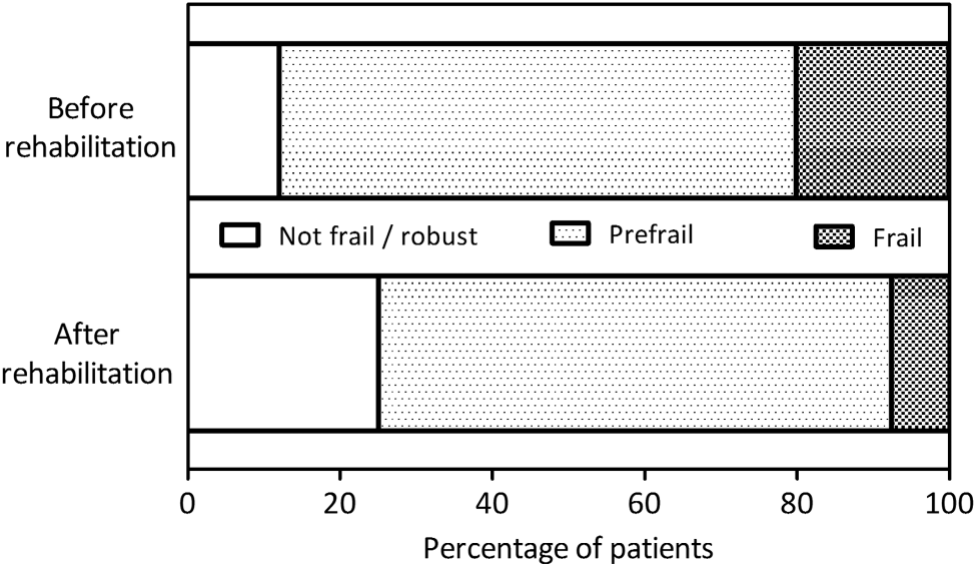
Patients with COPD grouped according to Fried's frailty criteria before and after pulmonary rehabilitation (n=574). Overall, rehabilitation led to a shift away from physical frailty towards a more robust state.

Following completion of rehabilitation, significant improvements were observed across all groups for SMI, QMVC, CRQ dyspnoea, fatigue, emotional and mastery domains, and physical activity (table 3) and in prefrail and frail groups for MRC score, handgrip strength, ISWT, CAT score and HADS domains (table 3). Adjusting for age and sex, a gradient of treatment response in favour of patients who were frail was evident for MRC score, handgrip strength, ISWT, CRQ fatigue, emotional and mastery domains, CAT score and HADS scores (table 3). Outcomes related to frailty characteristics (handgrip strength, 4MGS and physical activity) and responses to CES-D exhaustion questions also improved, such that postrehabilitation, fewer patients met case criteria for frailty (figure 3). Among the 115 completers who were frail prior to pulmonary rehabilitation, 71 (61.7%) were prefrail (64, 55.6%) or robust (7, 6.1%) following it. A small minority of completers, 13/390 (3.3%), had moved from a prefrail to a frail state (figure 3).

**Table 3:** Comparison of clinical outcomes following pulmonary rehabilitation according to frailty status

	Not frail (n=69)	Prefrail (n=390)	Frail (n=115)	p Value
MRC	0.1 (−0.3 to 0.5)	−0.5 (−0.7 to −0.4)	−1.4 (−1.1 to −1.7)*†	<0.001
SMI (kg/m^2^)	0.6 (0.5 to 1.1)	0.5 (0.2 to 0.7)	0.5 (0.1 to 0.8)	0.90
Handgrip (kg)	−0.2 (−1.2 to 0.9)	1.2 (0.8 to 1.5)	1.6 (1.0 to 2.3)*	0.002
Peak QMVC (kg)	2.7 (1.1 to 4.3)	1.9 (1.2 to 2.5)	1.8 (0.8 to 2.7)	0.55
Below QMVC cut-point (%)	6.4 (−1.4 to 14.1)	−21.74 (−17.7 to −25.3)	−36.6 (−24.8 to −46.9)*†	<0.001
4MGS (m/s)	0.08 (0.05 to 0.12)	0.07 (0.05 to 0.08)	0.11 (0.09 to 0.14)	0.004
ISWT (m)	17.8 (−21.7 to 57.3)	51.8 (24.4 to 79.2)	145.9 (108.6 to 183.2)*†	<0.001
CRQ dyspnoea score	3.8 (1.4 to 6.2)	4.4 (3.3 to 5.4)	6.8 (5.0 to 8.5)	0.006
CRQ fatigue score	−0.8 (−3.2 to 1.5)	3.1 (2.1 to 4.0)	6.1 (4.6 to 7.7)*†	<0.001
CRQ emotional score	−0.5 (−2.6 to 3.7)	4.0 (2.4 to 5.6)	8.6 (5.6 to 11.5)*†	<0.001
CRQ mastery score	0.7 (−1.1 to 2.4)	3.1 (2.1 to 4.1)	5.2 (3.4 to 6.9)*†	<0.001
Self-reported weekly energy expenditure (kcal)	1276.0 (714.1 to 1838.0)	606.2 (390.0 to 822.5)	767.1 (546.4 to 987.8)	0.08
Self-reported time in moderate activity (min/week)	417.5 (184.7 to 650.4)	137.0 (75.2 to 198.9)	190.3 (127.4 to 253.3)	0.006
CAT score	0.4 (−1.4 to 2.1)	−1.3 (−2.7 to 0.2)	−7.3 (−9.7 to −4.8)*†	<0.001
Katz score	0.0 (−0.1 to 0.1)	0.0 (−0.1 to 0.1)	0.1 (−0.1 to 0.3)	0.73
HADS anxiety	−0.3 (−2.0 to 1.4)	−1.0 (−1.7 to −0.3)	−2.8 (−4.4 to −1.2)*	<0.001
HADS depression	0.9 (−0.2 to 2.1)	−0.8 (−1.4 to −0.1)	−2.9 (−4.0 to −1.7)*†	<0.001

Values are mean change (95% CI) pre-to-post rehabilitation.

*Statistically different to non-frail group.

†Statistically different to prefrail group—tested if ANCOVA p value <0.003 following Bonferroni adjustment for multiple testing.

4MGS, 4-m gait speed; ANCOVA, analysis of covariance; BMI, body mass index; CAT, COPD Assessment Test; CRQ, Chronic Respiratory Disease Questionnaire; HADS, Hospital Anxiety and Depression scale; ISWT, incremental shuttle walk test; kcal, kilocalorie; MRC, Medical Research Council; QMVC, quadriceps maximum voluntary contraction; SMI, skeletal muscle mass index.

## Discussion

In this prospective cohort study, we identified that one-quarter (25.6%) of patients with stable COPD referred for pulmonary rehabilitation were frail according to the Fried phenotype criteria. Patients who were frail demonstrated high levels of impairment as compared with prefrail or robust patients, one consequence of which was more difficulty engaging in pulmonary rehabilitation as a mainstay of their disease management. Adjusting for all known confounders, being frail was associated with over double the odds of programme non-completion. Nonetheless, in those who completed rehabilitation there was a gradient of treatment response, in favour of patients who were frail, for outcomes relating to symptom burden, exercise performance and health status. Furthermore, in a large proportion of those who completed pulmonary rehabilitation, the frailty phenotype was reversed, at least in the short term.

Our point prevalence estimate for frailty is within the range previously reported in COPD (10.2% to 28.0%),^5^ ^7^ ^37^ and over twice the 9.9% (95% CI 9.6% to 10.2%) observed among older people living in the community.^2^ Prevalence in our cohort, with a mean age of 70 years, is similar to that found among those aged 85 and above in the general population.^2^ The only estimate to suggest frailty prevalence is not increased in respiratory disease arose from a retrospective analysis of an older-persons cohort and included patients with very mild spirometric disease (mean FEV_1_ 79.6 (25.2) % predicted) and minimal evidence of functional impairment (only 2.5% of patients with COPD had a slow gait speed).^5^ While frailty was more common in patients with spirometrically advanced disease, prevalence among patients in the GOLD I category was almost 20% emphasising the importance of multidimensional assessment, even in early disease.

The detailed phenotypic data highlight the extent to which frailty adversely impacts patients with chronic respiratory disease. Others have associated frailty with poor exercise capacity and physical disability.^7^ ^38^ We extend these findings by demonstrating that frailty relates to reduced physical function (lower limb muscle strength, exercise capacity, physical activity), dyspnoea, dependency, increased anxiety and depression, and worse emotional distress and health status. These deficits have also been linked to sarcopenia,^15^ which is considered a component of frailty.^39^ Frailty is more multifaceted and can occur without skeletal muscle dysfunction, for example, due to ventilatory impairment, as confirmed by the only partial overlap we observed between these two syndromes.^40^

Irrespective of cause, frailty denotes an increased state of risk related to falls, disability, hospitalisation and mortality.^3^ The prognostic utility of frailty in chronic respiratory disease for mortality and hospitalisation is supported by earlier studies,^5^ ^7^ ^41^ and its adverse impact on the clinical management of patients is emerging.^7^ Here, we highlight a new example of frailty altering COPD management in that it prevented patients from fully engaging in pulmonary rehabilitation, an internationally recommended standard of care.^11^ Patients who were frail experienced frequent episodes of clinical deterioration and/or hospitalisation, which restricted their participation in rehabilitation. Although previous studies have identified risk factors for pulmonary rehabilitation non-completion, for example, smoking status, breathlessness or low mood,^17^ ^18^ frailty was comparatively a far stronger independent predictor in our cohort.

The impressive outcomes seen following programme completion provide strong grounds to explore how better to support patients who are frail through rehabilitation, potentially through organisation changes or by supplementing supervised exercise with novel strategies, for example, neuromuscular electrical stimulation.^42^ Improvements across physical, psychological and global health were observed after rehabilitation, often with an apparent gradient of treatment response in favour of patients who were frail (table 2). The magnitude of effect among patients who were frail is noteworthy and many improvements far exceeded minimum important differences (MID); mean change (95% CI) ISWT 146 m (109 to 183)/MID 47.5 m^43^ and CAT score −7.3 (−9.7 to −4.8) MID −2.0.^34^ This was reflected in the shift away from frailty after rehabilitation shown in figure 3. In part, this reflects the working of the Fried phenotype model, which uses cut-points to define frailty, therefore in some patients a subtle improvement would ‘declassify’ their frail state. Nonetheless, our data validate the contemporary view of frailty as a condition that is amenable to treatment.^3^ ^39^ Treatments with evidence of efficacy in frailty management include exercise, nutritional support, self-management strategies and reduction of polypharmacy.^39^ Many of these components have already been operationalised to be delivered as pulmonary rehabilitation, which raises the idea the model could be adapted to support frail people outside of the respiratory specialty. Indeed, tailored frailty programmes for older adults are being piloted within geriatric medicine—the aims, structure and content of which are similar to pulmonary rehabilitation.^44^ We believe there is an opportunity to share learning, skills and component interventions to benefit patients across settings.

There are limitations to consider. We purposefully selected the Fried model which mainly reflects physical frailty and incorporates measures that are effort dependant or rely on patient recall. However, the model has proven construct and predictive validity and is the most established criterion measure of frailty.^3^ ^7^ ^9^ Other frailty assessments may capture the broader experience of the syndrome, for example, cognitive, social or environmental.^2^ We only enrolled patients who attended for pulmonary rehabilitation assessment, and there will likely be patients with ‘hidden’ frailty who were referred but unable to attend due to poor mobility or cognition. Our prevalence estimate does not take this subgroup into account, but this may be counterbalanced by the omission of asymptomatic patients with COPD, who may not have been referred to hospital outpatient or pulmonary rehabilitation clinics. We did not obtain outcomes on participants declining or dropping out of rehabilitation; therefore, our findings concerning clinical response to rehabilitation should not be generalised beyond those completing a programme. As patients who were frail had the greatest levels of impairment at baseline, regression to the mean may partially accounts for the preferential response. However, this bias is likely to be small and withholding rehabilitation from a control group could be considered unethical. The differences in some outcomes between patients who were frail and not-frail were, in part, a product of the poor response for the 10% of the cohort considered robust. This was most notable for exercise capacity and health-related quality of life, which may reflect that this fitter group had better preserved exercise capacity and health status at enrolment, and therefore had alternative targets for pulmonary rehabilitation. An example might be behavioural outcomes such as daily physical activity, which increased significantly in this group and to a greater extent to the prefrail and frail groups. Finally, outcomes were obtained immediately following rehabilitation and may reflect a transient change in frailty status. The value of these changes in COPD with respect to long-term outcomes, for example, admissions and mortality, will emerge in due course.

In conclusion, frailty affects one-quarter of patients with stable COPD assessed for pulmonary rehabilitation. Frailty is an independent risk factor for programme non-completion but appears to result in favourable rehabilitation outcomes. Future research should identify how best to support patients who are frail through pulmonary rehabilitation.
