# Supplementary material for: Physical frailty and pulmonary rehabilitation in COPD: a prospective cohort study
Source: Thorax. 2016 Jun 13;71(11):988–95. doi: 10.1136/thoraxjnl-2016-208460 (PMC5099190; doi:10.1136/thoraxjnl-2016-208460)
Supplement: Supplementary data [file thoraxjnl-2016-208460supp.pdf]

ONLINE SUPPLEMENTARY FILE

Physical frailty and pulmonary rehabilitation in COPD: a prospective cohort study.

Matthew Maddocks [1], Samantha S.C. Kon [2,3], Jane L. Canavan [2,4], Sarah E. Jones [2,4], Claire M. Nolan [2,4], Alex Labey [2,4], Michael I. Polkey [2], William D-C. Man [2,4]

TABLE S1: Fried model used to define frailty

**Characteristic**	**Assessment**	**Criteria**
Shrinking	Asked "In the last year, have you lost more than 10 pounds or ≥4.5 kilograms unintentionally (i.e. not due to dieting or exercise)?"	Responds "yes"
Exhaustion	Using the CES-D Depression Scale, the following two statements are read. (a) I felt that everything I did was an effort; (b) I could not get going.<br><br>Asked "How often in the last week did you feel this way?"<br><br>0 = rarely or none of the time (<1 day), 1 = some or a little of the time (1-2 days), 2 = a moderate amount of the time (3-4 days), or 3 = most of the time.	Responds "2" or "3" to either question.
Low physical activity	Asked using the modified Minnesota Leisure-time Physical Activity Questionnaire about activities in the last week. Kilocalories (Kcal) expended are calculated using a standardized algorithm.	Males <383 Kcal<br><br>Females <270 Kcal
Slowness	Four-metre gait speed test.	Males ≤173 cm in height: ≤0.762 m/s^2<br><br>Males >173 cm in height: ≤0.653 m/s^2<br><br>Females ≤159 cm in height: ≤0.762 m/s^2<br><br>Females >159 cm in height: ≤0.653 m/s^2
Weakness	Handgrip strength using a Jamar hand-held dynamometer. Maximum value over 3 tests on each hand, each separated by 30 seconds of rest.	Males BMI<24: ≤29kg<br><br>Males BMI 24.1-28: ≤30kg<br><br>Males BMI >28: ≤32kg<br><br>Females BMI<23: ≤17kg<br><br>Females BMI 23.1-26: ≤17.3kg

		Females BMI 26-29: ≤18kg<br><br>Females BMI >29: ≤21kg

Each characteristic is scored 0 or 1 if the criterion is met providing an overall score ranging 0-5. Using an ordinal scoring approach, patients with no criteria present are considered not-frail, those meeting 1-2 criteria are considered pre-frail, and those with ≥3 criteria present are considered frail.

TABLE S2: Characteristics of frail patients with COPD according to completion of pulmonary rehabilitation.

	Did not complete (n=94)	Completed (n=115)	p value
Age (years)	71.9 (11.3)	73.1 (8.6)	0.39
Male, n (%)	45 (47.9)	65 (56.5)	0.21
Smoking status current:former:never (%)	23.4: 70.2: 6.4	8.7: 82.6: 8.7	0.018
FEV1 % predicted	43.9 (19.7)	48.3 (20.4)	0.12
MRC score	4.3 (0.8)	3.9 (0.9)	0.001
Age-adjusted Charlson Score	4.1 (1.6)	4.3 (1.7)	0.89
ADO score	6.1 (1.7)	5.8 (1.4)	0.17
BMI (kg/m)	27.5 (7.7)	28.1 (7.5)	0.59
SMI (kg/m^2)	8.0 (1.9)	8.2 (1.8)	0.46
Sarcopenia (%)	25.6 (17.7, 35.4)	23.5 (16.7, 32.0)	0.81
Handgrip (kg)	20.4 (9.9)	21.9 (8.0)	0.20
Peak QMVC (kg)	20.1 (8.4)	21.6 (8.2)	0.26
QMVC % predicted	49.3 (18.3)	52.4 (15.8)	0.24
Below QMVC cut-point	48.6 (37.2, 60.0)	40.8 (31.6, 50.7)	0.32
4MGS (m/s)	0.64 (0.21)	0.68 (0.20)	0.14
ISWT (m)	89.4 (84.1)	117.6 (92.1)	0.023
CRQ dyspnoea score	13.0 (5.4)	12.4 (4.7)	0.34
CRQ fatigue score	11.2 (4.7)	10.6 (4.5)	0.32
CRQ emotional score	26.2 (8.9)	26.8 (10.0)	0.64
CRQ mastery score	14.4 (5.5)	15.5 (5.8)	0.18
Self-reported weekly energy expenditure (kcal)	179.8 (244.1)	320.5 (558.5)	0.024
Self-reported time in moderate activity (mins/week)	49.6 (67.5)	93.1 (160.9)	0.015
CAT score	25.3 (7.7)	24.7 (8.0)	0.60
KATZ score	5.4 (1.1)	5.5 (0.9)	0.26
HADS anxiety	8.7 (5.0)	8.0 (5.3)	0.34
HADS depression	8.4 (3.8)	8.1 (4.1)	0.58
Frailty characteristic (% meeting criteria)			
Unintentional weight loss	26.6 (18.7, 36.3)	24.3 (17.4, 32.9)	0.71
Exhaustion	85.1 (76.5, 90.9)	81.7 (73.7, 87.7)	0.52
Low physical activity	84.0 (75.3, 90.1)	77.4 (68.9, 84.1)	0.23
Slow gait speed	73.4 (63.7, 81.3)	71.3 (62.5, 78.8)	0.74
Weak handgrip strength	79.6 (70.3, 86.5)	81.7 (73.7, 87.7)	0.69

Values are mean (SD) or proportions (95% confidence interval). Legend: 4MGS = 4-metre gait speed, BMI = body mass index, CAT = COPD Assessment Test, CRQ = Chronic Respiratory Disease Questionnaire, FEV_1 – Forced expiratory volume in one second, HADS = Hospital Anxiety and Depression scale, ISWT = incremental shuttle walk test, kcal = kilocalorie, MRC = Medical Research Council, QMVC = quadriceps maximum voluntary contraction, SMI = skeletal muscle index.
